# Time-Restricted Eating and Its Metabolic Benefits

**DOI:** 10.3390/jcm12227007

**Published:** 2023-11-09

**Authors:** Sneha Mishra, Patress A. Persons, Andrea M. Lorenzo, Swarna S. Chaliki, Sophie Bersoux

**Affiliations:** Division of Community Internal Medicine Mayo Clinic, Scottsdale, AZ 85259, USA; persons.patress@mayo.edu (P.A.P.); lorenzo.andrea@mayo.edu (A.M.L.); chaliki.swarna@mayo.edu (S.S.C.); bersoux.sophie@mayo.edu (S.B.)

**Keywords:** circadian rhythm, fasting, lifestyle, time restricted, weight loss

## Abstract

Newer management strategies are being evaluated to treat obesity, which continues to increase worldwide. After 12 h of fasting, the body switches from glucose to fat metabolism, regulating protein synthesis and autophagy. These cellular responses are central to the metabolic benefits of time-restricted eating (TRE), independent of calorie restriction and weight loss, and they have heightened interest in TRE regimens. Controversy remains, however, regarding the benefits of TRE regimens. We reviewed the current literature and concluded that TRE is equivalent to calorie restriction for weight loss and has positive effects for patients with diseases such as nonalcoholic fatty liver disease, cancer, and cardiovascular disease.

## 1. Introduction

Obesity is a rapidly growing worldwide epidemic. The World Health Organization estimates that, by 2025, approximately 167 million people will become less healthy because they are obese or overweight [1]. Calorie restriction has always been the main approach to managing obesity and reducing cardiometabolic risk factors [2], although, for many persons, adhering to a program long term is difficult, making maintaining weight loss elusive. Periodic fasting for medical and religious reasons has been practiced since prehistoric times [3]. For example, millions of Muslims participate in a form of intermittent fasting during the month of Ramadan, when they fast from dawn to sunset. In the past decade, intermittent fasting regimens have become more popular as a way for patients to restrict calories with better adherence and to gain the metabolic benefits of these fasting regimens. However, debate has continued regarding their benefits and mechanisms of action. Therefore, we reviewed the current literature (PubMed and Cochrane) to define the most common types of intermittent fasting; describe the cellular response to fasting; and review the findings on health benefits, specifically in terms of obesity, diabetes, nonalcoholic fatty liver disease, cardiovascular disease, cancer, neurodegenerative disorders, sleep, and sarcopenia.

### 1.1. Intermittent Fasting Regimens

**Figure 1:**
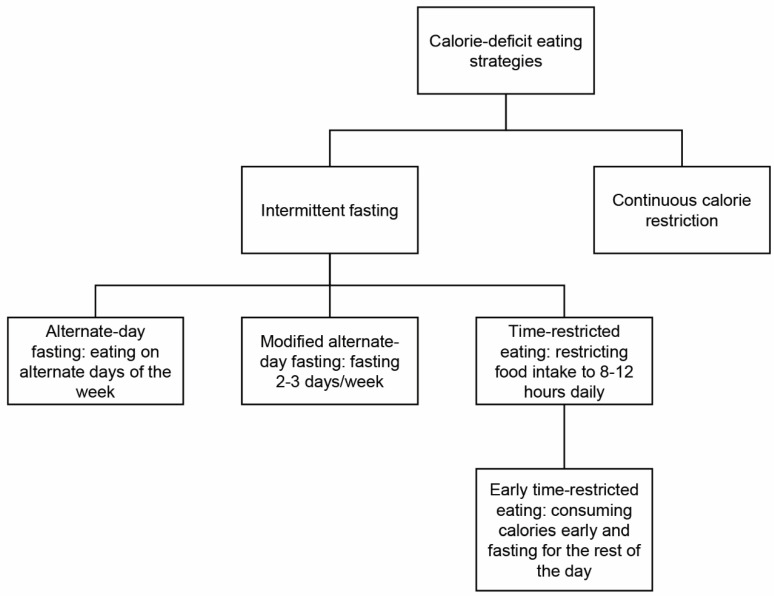
Types of calorie-deficit eating patterns.

The three most widely studied intermittent fasting regimens are alternate-day fasting (ADF); modified alternate-day fasting (MADF), e.g., 2 days per week (5:2 intermittent fasting); and daily time-restricted eating (TRE) (Figure 1). With TRE, a person restricts their food intake to 6 to 8 h each day [4]. This dietary strategy may help persons adhere better to restricting calories [5]. Early TRE (eTRE) is another intermittent fasting regimen. In eTRE, persons consume calories early in the day and fast for the remainder of the day. eTRE is time-restricted eating from 7 AM to 3 PM during the day. In a randomized control trial conducted by Jamshed et al., this was the time period used. For ADF and MADF, calorie intake varies from less than 25% of caloric needs to complete fasting for a day, alternating with nonfasting days without restrictions [6]. In the MADF regimen, intake during fasting days accounts for 20% to 30% of a normal dietary intake. This means that, on fasting days, individuals consume a reduced amount of calories compared to their usual intake; it is, however, not a complete fast where zero calories are consumed. This modification may make MADF more sustainable for some individuals than a strict zero-calorie ADF fasting regimen [7]. In an article published by Eshghiania et al. to study the effects of MADF on weight and CAD risk factor reduction in overweight and obese women, during the 6-week period of fasting, all subjects consumed a very-low-calorie diet on three fast days, which were Saturday, Monday, and Wednesday, and then they consumed their usual diet on the other days [8]. It is important to note that there are different variations of MADF, and the regimen used in this study is just one example. Different MADF regimens may involve varying degrees of calorie restriction on fasting days and different patterns of fasting and nonfasting days.

### 1.2. Cellular Responses to Fasting and Metabolic Switch

**Figure 2:**
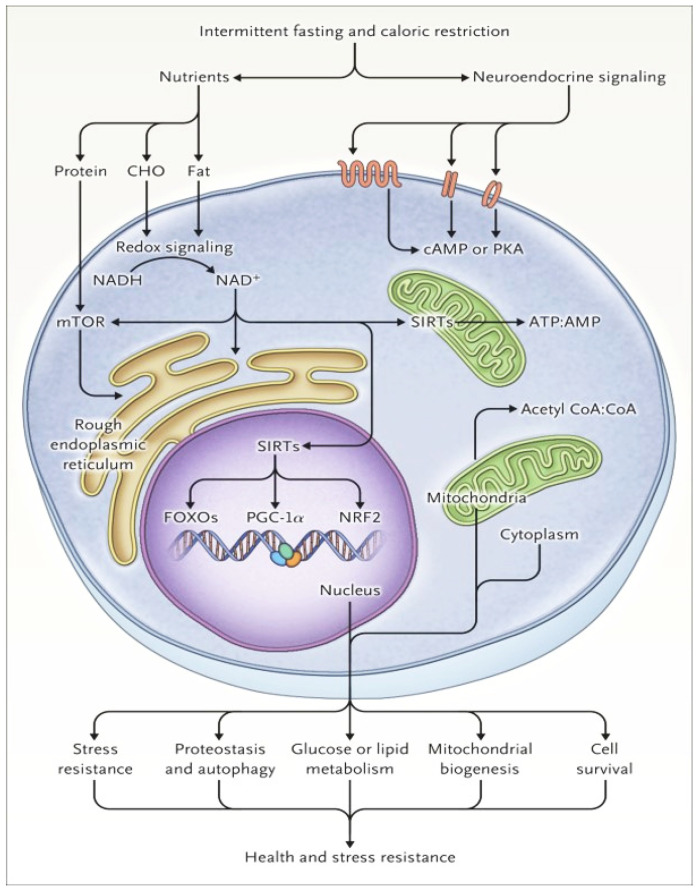
Cellular response to energy restriction. AMPK, AMP kinase; ATP, adenosine triphosphate; cAMP, cyclic AMP; CHO, carbohydrate; CoA, coenzyme A; FOXOs, forkhead box Os; IGF-1, insulin-like growth factor 1; mTOR, mammalian target of rapamycin; PGC-1α, peroxisome proliferator-activated receptor γ coactivator 1α; PKA, protein kinase A; NAD, nicotinamide adenine dinucleotide; NRF2, nuclear factor erythroid 2-related factor 2; redox, reduction–oxidation, SIRTS, sirtuins (from [11], used with permission).

The health benefits of fasting result from “flipping the metabolic switch”, as described by Anton et al. [9]. The change occurs after at least 12 h of fasting, when the body switches from using glucose as a fuel source to using ketone bodies. This change represents the point of negative energy balance, when the liver’s glycogen reserves are depleted and free fatty acids are mobilized, leading to fatty acid-derived ketone bodies. At the cellular level, ketone bodies enhance the pathways of the mammalian target of rapamycin (mTOR) and AMP-activated protein kinase [4]. In the mTOR pathway, cells regulate protein synthesis, activating on the basis of available nutrients and the person’s activity level. Changes resulting from the repeated challenges of metabolic shift lead to increased adaptation to stress, injury, and disease [3]. Moreover, ketone bodies increase mitochondrial stress resistance, antioxidant defenses, DNA repair, and autophagy. Autophagy is not cell death per se; nevertheless, autophagy has been reported to accompany cell death induced by nutrient starvation [10]. Decreased insulin levels, decreased mTOR, and less protein synthesis improve the resistance of cells and organs to stress, cell growth, and tissue remodeling, a setting that leads to increased resilience and disease resistance (Figure 2).

### 1.3. Effects of Time of Day on Obesity

Various groups have studied eating at different times of day to determine the effects of meal timing on obesity and cardiometabolic health. Because the circadian rhythm, insulin sensitivity, and the thermic effect of food peak in the morning, eTRE may offer more benefits than other forms of TRE [12]. In a study comparing meal timing (three meals) for women with metabolic syndrome, breakfast vs. dinner as the primary meal resulted in greater weight loss, reduced waist circumference, improved fasting glucose, decreased insulin resistance, decreased ghrelin and insulin levels, and higher rates of satiety in the group eating breakfast as the primary meal [13]. A 5-week randomized crossover trial of eTRE in men with prediabetes showed improved insulin levels, insulin sensitivity, blood pressure, and oxidative stress for the eTRE group, even though the participants received enough food to maintain their weight, and no weight loss occurred [14]. Bonham et al. [15] compared the obesity rates of day vs. night shift workers in a systematic review and meta-analysis. They concluded that night shift workers had an increased rate of obesity despite there being no significant difference between the groups in reported energy intake. This finding suggests that other factors such as the circadian rhythm, the timing of meals, and differences in nighttime energy metabolism may be responsible for the increased obesity rate.

The Healthy Heroes randomized controlled trial showed decreased levels of glycated hemoglobin A1c (HbA1c), a lower diastolic blood pressure, and a significantly decreased particle size of very-low-density lipoprotein in 24 h shift workers than in a standard diet group [16].

### 1.4. Effects of TRE on Health

#### Obesity

TRE fosters weight loss mainly because of the reduced energy intake (10–30%) [17]. Trials have aimed to determine the effects of TRE on weight loss, but the findings have been inconsistent. For example, the TREAT (Time-Restricted EAT) randomized trial was designed to study weight loss and other metabolic parameters in women and men with overweight and obesity (112 participants over 12 weeks) [18]. The findings showed that TRE, in the absence of other interventions, was no more effective for weight loss than continuous calorie restriction. The results of a systematic review of 27 trials reporting on intermittent fasting showed that participants who fasted had a weight loss of 0.8% to 13% without serious adverse effects [19]. Calorie restriction and intermittent fasting had equivalent results in this study. In a randomized controlled trial, patients following eTRE (8 h window) lost more weight than patients eating during a window of at least 12 h [20]. These results have led us to believe that TRE carried out to match the body’s circadian rhythm is a more effective strategy for weight loss.

A review of the literature suggests that TRE is a feasible strategy. With TRE, persons do not have to count calories regularly but may still take in fewer calories by decreasing the time interval during which they can consume food [2]. Moreover, TRE can be modified to align with a patient’s existing dietary routine and preferences, which may lead to better patient compliance [21]. A randomized controlled trial reported that participants adhered to TRE for an average of 6 days per week, and TRE resulted in improved mood, better vigor, and less depression, which further enhanced adherence to the plan as a lifestyle strategy [20]. A network meta-analysis of 12 randomized controlled trials involving 730 participants suggested that both early and later time-restricted eating (TRE) can effectively reduce body weight gain and improve insulin resistance (HOMA-IR) compared to non-TRE approaches. Notably, early TRE appears to be more beneficial for enhancing insulin sensitivity and glycemic metabolism and is associated with lower blood pressure when compared to non-TRE. While early TRE shows a trend toward greater weight loss than later TRE, this difference is not statistically significant. The findings support TRE as a viable and practical weight management strategy, with early TRE potentially offering additional metabolic advantages. However, further large-scale studies are needed to validate these results and to assess the long-term effects [22].

## 2. Clinical Pearl: eTRE Carried Out to Match the Body’s Circadian Rhythm Is More Effective for Weight Loss

### 2.1. Diabetes

Sutton et al. [14] reported that eTRE in a 6 h window with dinner before 3:00 PM improved the insulin resistance of patients with prediabetes and type 2 diabetes, independent of the weight loss benefit. The mechanism of action appeared to be related to fatty acid mobilization, β-oxidation, enhanced ketone body production, and the induction of autophagy [9]. In a randomized controlled trial designed to evaluate the effects of TRE in overweight patients with type 2 diabetes, a 10 h TRE window decreased the mean daily calorie intake by 28% compared with the intake of a control group [23]. All participants completed the 12-week study, and no adverse effects were reported (e.g., headaches, thirst, or diarrhea). Fasting blood glucose decreased by 18%, and no hypoglycemic events occurred in the TRE group. However, in a 12-month, randomized controlled trial comparing ADF, daily calorie restriction (controlled diet), and a no-intervention control, participants in both intervention groups lost weight, but no significant change occurred in insulin sensitivity or HbA1c compared with the no-intervention control group [24]. The INTERFAST (Intermittent Fasting)–2 trial evaluated the efficacy and safety of intermittent fasting for patients with insulin-treated type 2 diabetes and showed that ADF for 12 weeks improved HbA1c, reduced body weight, and led to a total daily decrease in insulin dose [25].

Clinical Pearl: eTRE Helps Improve Insulin Resistance Independent of the Weight Loss Benefit

### 2.2. Nonalcoholic Fatty Liver Disease

TRE appears to be safe and effective in nonalcoholic fatty liver disease (NAFLD). In prolonged fasting, ketone bodies not only become the primary source of energy but also play an important role at the cellular level, as described above, leading to improved lipolysis. The metabolic benefits to the liver are independent of weight loss and calorie restriction. Human studies on NAFLD and TRE are scarce, and most data have been obtained from studies conducted during the Ramadan holiday, when fasting is carried out for 12 to 14 daylight hours for 30 days. However, the food consumed during the Ramadan holiday may have a higher sugar and fat content and, thus, may not be representative of a usual diet. Even so, studies have reported weight loss and improvement in noninvasive markers of liver fibrosis, such as the fibrosis-4 score, insulin resistance, and inflammatory markers, compared with controls [26]. Further studies are needed to determine the benefit of intermittent fasting compared with and/or combined with diets such as the well-established, reproducible Mediterranean diet.

Clinical Pearl: TRE is a safe and efficacious method for improving fibrosis via weight-loss-dependent and weight-loss-independent mechanisms, including shifting metabolic processes away from hepatic lipogenesis, and for improving insulin resistance and metabolic syndrome.

### 2.3. Cardiovascular Diseases

TRE may potentially improve cardiovascular health. Although more studies are needed, the available studies show possible benefits of TRE for blood pressure and the lipid profile. In a meta-analysis of 19 studies, TRE lowered systolic blood pressure in 6 of the studies (a total of 97 participants) [27]. In another recent study with 90 participants, eTRE combined with calorie restriction lowered diastolic blood pressure by 4 mm Hg compared with a control group following calorie restriction alone, although no statistically significant differences in systolic blood pressure were reported [20]. The effects of Ramadan fasting on cardiometabolic risk factors in healthy adults were reported in a systematic review and meta-analysis of 91 studies involving 4431 adults [28]. In this analysis, fasting improved cardiometabolic risk factors because many of the included studies showed at least a transient decrease in serum triglyceride, total cholesterol, and low-density lipoprotein levels and an increase in high-density lipoprotein levels.

Clinical Pearl: TRE improved cardiometabolic risk factors by decreasing serum triglyceride, total cholesterol, and LDL cholesterol levels. TRE combined with calorie restriction is more efficacious for diastolic blood pressure control than calorie restriction.

### 2.4. Cancer

Obesity and metabolic syndrome have been associated with an increased incidence of cancer, including colorectal, kidney, esophageal, endometrial, breast, pancreatic, thyroid, liver, ovarian, gallbladder, and prostate cancers, and non-Hodgkin lymphoma [29]. For patients diagnosed with cancer, obesity worsens the prognosis, increases the risk of recurrence, and decreases survival. In 2014, the American Cancer Society reported that 7.8% of oral cancers and 6.5% of all cancer deaths in the US were attributed to excess body weight [30]. TRE has been shown to increase the anticancer activity of chemotherapeutic agents, such as tamoxifen and fulvestrant, and to delay resistance to these agents. In combination, TRE contributes to tumor regression and to reversing acquired resistance to these two chemotherapeutic agents [31]. In obesity, elevated cytokine levels cause chronic tissue inflammation and immune cell infiltration [32], and the resultant changes create a microenvironment that may initiate or accelerate cancer [33]. Whether proinflammatory markers decrease after TRE has not been shown conclusively. Proinflammatory markers decreased significantly after TRE in an 8 h window and after longer nighttime fasting in a study by Moro et al. [34], whereas, in a study by Cienfuegos et al. [17], these markers did not change.

Tumor growth has also been reported after the circadian rhythm is disrupted, a result of the dysregulation of key cell cycles and tumor suppressor genes [35]. Antitumor activity may be increased by decreasing the levels of insulin, insulin-like growth factor 1, and leptin via a fasting diet, such as TRE [31]. The potential link between shift work and an increased risk of breast cancer further illustrates this relationship with a disrupted circadian rhythm [36]. eTRE may also lower the risk of breast and prostate cancers [37]. The anticancer benefits of TRE include improved cellular health and activation of cell signaling pathways and integrative adaptive responses between and within organs that increase the expression of antioxidants, repair DNA, help maintain protein homeostasis under acute stress conditions, and promote mitochondrial biogenesis and autophagy. Repeated fasting has been shown to reduce cell proliferation, cancer progression, and metastases [38].

Clinical Pearl: TRE has been shown to increase the activity of chemotherapeutic agents, such as tamoxifen and fulvestrant. Repeated fasting has been shown to reduce cell proliferation, cancer progression, and metastases by maintaining protein homeostasis and promoting mitochondrial biogenesis and autophagy.

### 2.5. Neurodegenerative Diseases

Ezzati et al. [39] summarized the evidence on associations between obesity and the risk of cognitive decline, including Alzheimer disease and vascular dementia. TRE has been reported to improve cognitive function through various mechanisms, including by regulating the circadian rhythm and increasing autophagy, leading to decreased neuroinflammation, decreased oxidative stress, and increased insulin sensitivity [40]. Changes in the metabolic pathway (lipid synthesis and storage to the mobilization of fat through fatty acid oxidation and fatty acid-derived ketones) and weight loss were also reported to independently improve cerebrovascular function and cognitive function [41]. Mitochondrial dysfunction has been associated with cognitive impairments in studies of the aging brain and neurodegenerative diseases, and it may act by enhancing mitochondrial biogenesis and by regulating mitochondrial dynamics [42]. Therefore, TRE may be a strategy for improving mitochondrial function to delay neurodegenerative disease and improve longevity.

Clinical Pearl: Changes in the metabolic pathway (from lipid synthesis and storage to the mobilization of fat through fatty acid oxidation and fatty acid-derived ketones) and weight loss were reported to improve mitochondrial function. They may help delay neurodegenerative disease and improve longevity.

### 2.6. Sleep

Studies about the effects of intermittent fasting on sleep had largely discrepant results and used different methods and types of analyses. Several studies suggested that intermittent fasting can improve sleep quality, duration, and the latency of onset [43,44]. For example, persons practicing TRE or ADF had improved deep sleep and REM (rapid eye movement) sleep and fewer sleep disturbances [44]. These improvements may be attributed to various hormonal and metabolic responses, insulin sensitivity, and the regulation of the melatonin and circadian rhythms [45], although a prospective observational study did not show sleep duration to correlate with melatonin levels [46]. Persons using eTRE with an eating window between 07:00 and 15:00 also slept less and took longer to fall asleep, although they did not report poor sleep quality or fatigue [47]. A multisite, prospective cohort study of eTRE did not report an improved circadian rhythm for 547 participants with a wide range of body weights [48]. Larger studies with longer follow-ups are needed before concrete conclusions about intermittent fasting and sleep can be made.

Clinical Pearl: Most studies showed a negative or no impact on sleep with TRE.

### 2.7. Sarcopenia

TRE and resistance training appear to have a synergistic effect on preserving muscle mass and improving body composition by controlling blood glucose levels and reducing fat while increasing lean muscle mass, especially in overweight and obese adults [49]. An 8-week study comparing resistance training in normal eating and overweight and obese TRE participants showed a significantly reduced total body mass, body mass index, and fat mass in the TRE group; lean mass increased in both groups [49]. Adding moderate- to high-intensity exercise to TRE potentiates the metabolic switch through the accelerated depletion of glycogen in the liver [50].

Kotarsky et al. [49] reported equivalent maintenance or increased lean mass and skeletal muscle thickness in middle-aged and younger adults who exercise and practice TRE compared with persons not practicing TRE who had an equivalent protein intake and a similar energy intake. Fat mass has also been reported to decrease in resistance-trained men who practice TRE [51]. However, the effects of TRE are not well understood in adults 65 years or older. The primary anabolic stimuli that help preserve or increase skeletal muscle mass with aging are exercise and consistent protein intake throughout the day [52], which is contrary to the principles of TRE, wherein all calories are consumed in a restricted window of time. The anabolic response to protein ingestion is also attenuated in older vs. younger adults [52]. Additional studies are needed that focus on the potential benefit of TRE on muscle mass, function, and efficiency in populations of various ages.

Clinical Pearl: TRE and resistance training appear to have a synergistic effect on preserving and improving lean muscle mass while also controlling blood glucose levels and reducing fat.

Please see the table below representing our key findings (Table 1).

Metabolic conditions that did not significantly benefit from TRE: (Table 1).

This article sheds light on the potential benefits of time-restricted eating (TRE) across diverse metabolic conditions. However, it is crucial to acknowledge specific areas that require further investigation before drawing definitive clinical takeaways. One such area is the application of TRE to neurodegenerative diseases, which this article suggests may improve cognitive function and delay disease onset. Given the complex nature of these conditions, more comprehensive research is needed to understand the precise mechanisms and long-term effects of TRE in the context of neurodegenerative diseases. Additionally, TRE’s potential impact on metabolic conditions like MAFLD necessitates further exploration to determine the most effective and safe approaches for individuals with MAFLD. Furthermore, the varying results regarding the effects of TRE on sleep quality, with some studies suggesting improvements and others showing no significant impact, underscore the need for more research to elucidate the relationship between TRE and sleep, including its potential benefits and limitations. In summary, while TRE shows promise in various metabolic conditions, including neurodegenerative diseases, MAFLD, and sleep, it is imperative to approach these areas with caution and await more comprehensive investigations to establish specific clinical recommendations and fully grasp the potential benefits and limitations of TRE.

## 3. Discussion

The findings of our review suggest that TRE may have metabolic benefits that are associated with the change from glucose to fatty acid-derived ketones as the source of energy. This change triggers a shift from lipid storage to the mobilization of fat leading to fatty acid oxidation, which further preserves muscle mass. Even if weight loss from TRE does not prove to be superior to overall calorie restriction, TRE may be a good lifestyle strategy for persons struggling to control calories because it allows patients flexibility, as they are able to align the TRE schedule with their current diet, work, exercise, and sleep patterns, and it does not require calorie counting. In addition, TRE combined with exercise and resistance training may help older adults lose unhealthy fat while preserving muscle mass. When providing recommendations for TRE, clinicians may want to encourage patients to consume a heart-healthy diet, such as the Mediterranean diet or a plant-based diet, in addition to providing guidance on fasting windows. Patients should be encouraged to follow a plant-based diet and consume fruits and vegetables along with whole grains to improve the fiber and micronutrient intake of their diet. This article sheds light on the potential benefits of time-restricted eating (TRE) across diverse metabolic conditions. However, it is crucial to acknowledge specific areas that require further investigation before drawing definitive clinical takeaways. One such area is the application of TRE to neurodegenerative diseases, which this article suggests may improve cognitive function and delay disease onset. Given the complex nature of these conditions, more comprehensive research is needed to understand the precise mechanisms and long-term effects of TRE in the context of neurodegenerative diseases. Additionally, TRE’s potential impact on metabolic conditions like MAFLD necessitates further exploration to determine the most effective and safe approaches for individuals with MAFLD. Furthermore, the varying results regarding the effects of TRE on sleep quality, with some studies suggesting improvements and others showing no significant impact, underscore the need for more research to elucidate the relationship between TRE and sleep, including its potential benefits and limitations. This review has limitations, including the different terms used for intermittent fasting that made direct comparisons between studies difficult, studies that are not heterogeneous in design and approach, and protocols that are not consistent for metabolic switching. Future studies should be considered that use a metabolic switch biomarker, such as serum ketones, to measure compliance and assess negative energy balance. A limitation of this study is the omission of a comprehensive assessment of the quality of the included studies, which restricts the ability to gauge the reliability and validity of the results. Conducting a systematic evaluation of study quality would have provided valuable insights into the rigor and potential biases of the included randomized controlled trials, enhancing the interpretation of the findings.

In conclusion, TRE appears to be a safe method for providing metabolic benefits for many health conditions. Randomized trials that evaluate the various types of intermittent fasting in diverse populations are needed. Trials should also evaluate the Mediterranean diet and optimal sleep patterns along with TRE to determine what types of diets and fasting provide the best benefits for patients.

## Figures and Tables

**Table 1:** Metabolic effects of TRE.

Metabolic Condition	TRE Effects	Comments
Weight Loss	Promotes weight loss through reduced calorie intake within the restricted eating window.	The article mentions variable outcomes in different studies, suggesting the need for further investigation.
Diabetes (Prediabetes and Type 2)	Improves insulin resistance independent of weight loss. Enhances fatty acid mobilization, β oxidation, and ketone body production. Induces autophagy.	TRE shows promise in improving insulin sensitivity and glycemic control in prediabetes and type 2 diabetes patients.
NAFLD (Nonalcoholic Fatty Liver Disease)	Shifts metabolic processes away from hepatic lipogenesis. Improves insulin resistance and metabolic syndrome.	While human studies on TRE and NAFLD are limited, TRE appears to benefit liver health, potentially independent of weight loss.
Cardiovascular Health	May lower blood pressure and improve lipid profiles. Possible benefits for cardiometabolic risk factors.	The evidence suggests potential positive effects on cardiovascular health, but further research is required for definitive conclusions.
Cancer	Increases anticancer activity of certain chemotherapeutic agents. May inhibit tumor growth.	TRE, in combination with chemotherapy, has shown potential benefits in enhancing the efficacy of cancer treatment and inhibiting tumor growth.
Neurodegenerative Diseases	May improve cognitive function through circadian rhythm regulation and reduced neuroinflammation.	The relationship between TRE and neurodegenerative diseases is still evolving, necessitating more comprehensive research.
Sleep	Mixed results in studies, with some suggesting improvements in sleep quality.	The impact of TRE on sleep quality varies in different studies, requiring further investigation for a conclusive assessment.
Sarcopenia	May preserve lean muscle mass and improve body composition when combined with resistance training.	Combining TRE with resistance training shows potential for preserving lean muscle mass, especially in overweight and obese adults.

## Data Availability

Data sharing not applicable. No new data were created or analyzed in this study.

## Abbreviations

ADF	alternate-day fasting
eTRE	early TRE
HbA1c	hemoglobin A1c
MADF	modified alternate-day fasting
mTOR	mammalian target of rapamycin
NAFLD	nonalcoholic fatty liver disease
TRE	time-restricted eating
TREAT	Time-Restricted EAT
